# Antisense RNAs during early vertebrate development are divided in groups with distinct features

**DOI:** 10.1101/gr.262964.120

**Published:** 2021-06

**Authors:** Sanjana Pillay, Hazuki Takahashi, Piero Carninci, Aditi Kanhere

**Affiliations:** 1 Department of Cell, Developmental and Regenerative Biology, Mount Sinai School of Medicine, New York, New York 10029, USA;; 2 Laboratory for Transcriptome Technology, RIKEN Center for Integrative Medical Sciences, Yokohama, Kanagawa, 230-0045, Japan;; 3 Fondazione Human Technopole, 20157 Milan, Italy;; 4 Institute of Systems, Molecular and Integrative Biology, University of Liverpool, Liverpool, L69 3GE, United Kingdom

## Abstract

Long noncoding RNAs or lncRNAs are a class of non-protein-coding RNAs that are >200 nt in length. Almost 50% of lncRNAs during zebrafish development are transcribed in an antisense direction to a protein-coding gene. However, the role of these natural antisense transcripts (NATs) during development remains enigmatic. To understand NATs in early vertebrate development, we took a computational biology approach and analyzed existing as well as novel data sets. Our analysis indicates that zebrafish NATs can be divided into two major classes based on their coexpression patterns with respect to the overlapping protein-coding genes. Group 1 NATs have characteristics similar to maternally deposited RNAs in that their levels decrease as development progresses. Group 1 NAT levels are negatively correlated with that of overlapping sense-strand protein-coding genes. Conversely, Group 2 NATs are coexpressed with overlapping protein-coding genes. In contrast to Group 1, which is enriched in genes involved in developmental pathways, Group 2 protein-coding genes are enriched in housekeeping functions. Group 1 NATs also show larger overlap and higher complementarity with the sense-strand mRNAs compared to other NATs. In addition, our transcriptomics data, quantifying RNA levels from cytoplasmic and nuclear compartments, indicates that Group 1 NATs are more abundant in the cytosol. Based on their expression pattern, cytosolic nature, and their higher complementarity to the overlapping developmental mRNAs, we speculate that Group 1 NATs function post-transcriptionally to silence spurious expression of developmental genes.

Natural antisense transcripts (NATs) are noncoding RNAs that are transcribed in an antisense direction to the overlapping genes. NATs are prevalent in eukaryotic genomes, ranging from yeast to humans. Estimates suggest that, in the human genome, as many as ∼38% of protein-coding genes show evidence of antisense transcription (Katayama et al. 2005; He et al. 2008; Balbin et al. 2015). Although the importance of the majority of NATs still remains enigmatic, expression of sense-antisense transcript pairs is often linked, either positively or negatively, much more than expected by chance (Chen et al. 2005). Although the possibility that the antisense transcription is a mere consequence of transcriptional status of the overlapping protein-coding gene cannot be ruled out, a number of studies also suggest that there is a regulatory relationship between the expression of overlapping sense-antisense pairs. Individual examples suggest that NATs can regulate the overlapping protein-coding partner using a variety of transcriptional and post-transcriptional mechanisms, such as influencing chromatin landscape (Nagano et al. 2008; Wang et al. 2011; Magistri et al. 2012; Fatica and Bozzoni 2014), genomic imprinting (Berteaux et al. 2008; Zhao et al. 2008; Fatica and Bozzoni 2014), RNA processing (Mandal et al. 2013), alternative splicing (Morrissy et al. 2011), RNA stability, and the rate of translation (Beltran et al. 2008; Ebralidze et al. 2008). NATs such as *Kcnq1ot1* (Pandey et al. 2008; Fatica and Bozzoni 2014), *Airn* (Nagano et al. 2008), and *HOTTIP* (Wang et al. 2011) regulate transcription of the overlapping genes by influencing chromatin environment at the genomic locus. On the one hand, NATs like *HTT-AS* and *qrf* use transcriptional interference to down-regulate the expression of overlapping genes (Chung et al. 2011; Xue et al. 2014). Some NATs can also function post-transcriptionally, for example, by increasing the stability of overlapping sense-strand mRNAs by protecting them from RNA-degrading machineries such as microRNAs and ribonucleases. Antisense RNAs overlapping *Sirt1* and *BACE1* genes are good examples of this mechanism (Faghihi et al. 2008; Li et al. 2018). On the other hand, some NATs are shown to decrease the stability of overlapping sense-strand mRNA. In these cases, by virtue of sequence complementarity, NAT and sense-strand mRNA can hybridize resulting in inhibition of mRNA translation and leading to its degradation (Faghihi and Wahlestedt 2009; Villegas and Zaphiropoulos 2015). Finally, NATs like *THRA* NAT participate in regulating alternative splicing of overlapping *THRA* mRNA (Hastings et al. 2000).

Recent studies suggest that NATs play an important role during development. For example, NATs such as *Kcnq1ot1* (Pandey et al. 2008) and *Airn* (Nagano et al. 2008; Fatica and Bozzoni 2014) are needed for imprinting, which is crucial for early vertebrate development (Wang and Chang 2011). NATs like *HOTTIP* and *HOTAIRM1* are involved in regulation of spatiotemporal expression of developmentally important *HOXA* genes (Wang and Chang 2011; Wang et al. 2011). In addition, studies in animal models indicate that NATs play a role in embryonic development in vertebrates. Previous transcriptomics studies have identified large number of lncRNAs during embryonic development in vertebrates such as zebrafish (Ulitsky et al. 2011; Pauli et al. 2012; Haque et al. 2014). Up to 50% of maternally deposited RNAs in zebrafish embryo are NATs (Pauli et al. 2011, 2012). A transcriptomics study by Pauli et al. (2012), for example, identified 1133 lncRNAs across eight stages of zebrafish development; of these, 397 were intergenic lncRNAs, 184 intronic overlapping lncRNAs, and the rest 566 were classified as exonic overlapping NATs. The abundance of NATs during early development combined with functional studies on selected NATs further point to their importance in early embryonic development (Li et al. 2010; Pauli et al. 2012; Wei et al. 2014). However, for the majority of NATs, functional mechanism and relationship to the overlapping protein-coding transcript during development remains largely unclear (de Hoon et al. 2015).

NATs and their regulatory mechanisms during development are likely to be conserved across different vertebrates. *spi1b* NAT and *lnc.tie1* are perfect examples of this (Li et al. 2010; Wei et al. 2014). *lnc.tie1* is transcribed in zebrafish, mouse, and humans in the antisense direction to the *tie1* gene (Li et al. 2010). Tie1 protein is a tyrosine kinase receptor for angioproteins and is essential for vascular development in vertebrates. The NAT, *lnc.tie1*, binds to *tie1* mRNA through RNA:RNA hybridization and down-regulates it, thus resulting in a loss of the protein. This mechanism is evolutionarily conserved because imbalance in regulation of Tie1 protein by *lnc.tie1* results in vascular defects in zebrafish as well as human (Li et al. 2010). Another example is that of *spi1b* NAT, which regulates the expression of transcription factor Spib that regulates myeloid and lymphoid cell development in both zebrafish and human (Wei et al. 2014). Similar to *lnc.tie1*, *spi1b* NAT also down-regulates *spi1b* mRNA by forming an RNA–RNA duplex and preventing its translation.

Zebrafish is one of the popular animal models that is routinely used to understand early vertebrate development (Mork and Crump 2015; Drummond and Davidson 2016; Jung et al. 2017). In zebrafish, embryonic development starts by fertilization of externally laid eggs and spans across a period of three days post-fertilization (dpf). Initially, the embryo undergoes 10 rapid and asynchronous cell divisions followed by more lengthened cell cycles. In all vertebrates, the embryo is in a transcriptionally inactive state during the initial period of cell divisions. As a result, during this period when the zygotic genome is inactive, early development of the embryo is completely dependent on maternally provided products (Tadros and Lipshitz 2009). As development progresses, the transcription of the zygotic genome is activated and simultaneous clearance of maternal RNAs and proteins leads to their replacement with newly synthesized zygotic RNAs. This process is called maternal-to-zygotic transition (MZT). In zebrafish, MZT coincides with midblastula transition (MBT) and occurs at three hours post-fertilization (hpf) at the 1000-cell stage (Lee et al. 2014). The availability of a large amount of transcriptomics data during developmental stages spanning MZT makes zebrafish a good model to further interrogate relationship between NATs and their sense protein-coding partners during early development.

Unlike many other classes of ncRNAs, NATs show varied relationship with overlapping protein-coding genes. They also differ in size, their genomic organization, conservation, expression pattern, and cellular localization. The lack of any unifying characteristics or relationship between NATs and their overlapping protein-coding genes makes it difficult to assess their role in regulating protein-coding genes. This is also one of the reasons why the role of NATs during early vertebrate development remains enigmatic. Here, we present results of transcriptomics analyses of different groups of NATs during early zebrafish developmental stages spanning MZT. The aim of this study is to identify distinct features of each group of NATs, which will be useful in speculating their role in development and in predicting their function in regulating sense-strand gene expression.

## Results

### Comparison of NATs with long intergenic RNAs during early development

First, we sought to understand if NATs have features that are distinct from other long ncRNAs. We compared them to the other major category of lncRNAs, long intergenic ncRNAs (lincRNAs). LincRNAs, unlike NATs, are expressed from genomic loci that are away from the protein-coding regions of the genome. We first analyzed expression levels of NATs and lincRNAs in the eight stages of early development (2–4 cells, 1000 cells, dome, shield, bud, 24 hpf, 48 hpf, and 120 hpf). For this purpose, RNA-seq reads from a previously published study (Pauli et al. 2012) were remapped to the zebrafish genome, and a normalized abundance of annotated noncoding RNAs and protein-coding genes was calculated.

**Figure 1.**
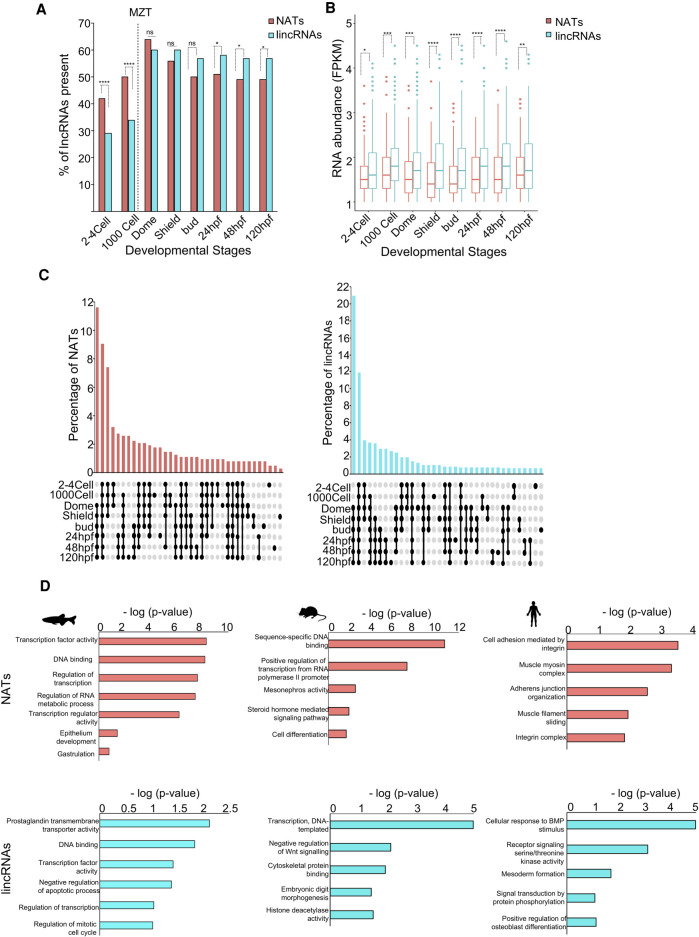
LncRNA dynamics during zebrafish development. (*A*) A bar plot showing the percentage of antisense and lincRNAs present during eight stages of zebrafish development. The significance values are as follows: (*) *P* < 0.05; (**) *P* < 0.005; (***) *P* < 0.001; (****) *P* < 0.0001; (ns) nonsignificant (χ^2^ test). (*B*) A box plot of abundance levels of NATs and lincRNAs across eight developmental stages. Each box shows median abundance value (as horizontal lines) and extend from 25th to 75th percentile values for each group. The outliers are shown as dots. The significance values are as follows: (*) *P* < 0.05; (**) *P* < 0.005; (***) *P* < 0.001; (****) *P* < 0.0001; (ns) nonsignificant (unpaired, two-tailed *t*-test). (*C*) UpSet diagrams depicting the percentage of NATs (*left*) and lincRNAs (*right*) that are common or unique in the eight stages of zebrafish development. The percentage of lncRNAs is shown on the *y*-axis, and the stages in which the lncRNA is present is shown *below* the *x*-axis. Filled circles represent the stages under consideration for the bar *above*. (*D*) Bar plots showing Gene Ontology terms associated with the mRNAs that overlap NATs (*top*) versus mRNAs neighboring to the lincRNAs (*bottom*) in zebrafish, mouse, and human. The −log(*P*-value) values for gene enrichment are plotted on the *x*-axis, and Gene Ontology terms are shown on the *y*-axis.

We first compared the percentage of annotated NATs and lincRNAs that were expressed (>1 FPKM) during different stages of development (Fig. 1A). The percentage of NATs detected during maternal stages (2–4 cells and 1000 cells) was higher than the percentage lincRNAs (Fig. 1A). However, after zygotic genome activation, the percentage of NATs was lower than lincRNAs. Before MZT, NAT percentages (∼13% in the 2- to 4-cell stage and 15% in the 1000-cell stage) were significantly higher than that of lincRNAs (0.2%–3.5%, *P*-value < 10^−4^). On the other hand, after MZT, the percentage of NATs was significantly lower compared to lincRNAs (Fig. 1A). This indicates that a higher percentage of NATs is deposited among maternal RNAs than lincRNAs, suggesting possible relevance of NATs in the pre-MZT stages.

In addition, we also examined expression levels of NATs and lincRNAs. In all the eight stages that were considered in this study, the average RNA levels of NATs were lower (*P*-value < 0.01) compared to lincRNAs (Fig. 1B). This suggests that although types of NAT species present in the pre-MZT was more than lincRNAs (Fig. 1A), they were on an average less abundant than lincRNAs (Fig. 1B).

We also inspected the stability and stage specificity of these two classes of lncRNAs using UpSet diagrams (Lex et al. 2014). This allowed us to visualize the frequency of NATs and lincRNAs that were present in consecutive stages of development (Fig. 1C; Supplemental Table S1). This analysis indicated that the percentage of NATs stable during the first four stages spanning MZT was higher than lincRNAs (7.5% vs. 3.9%; χ^2^ *P*-value < 0.01). On the one hand, the percentage of NATs present in the six post-MZT stages was almost half that of the lincRNAs (11.6% vs. 21%; χ^2^ *P*-value < 0.0001). On the other hand, the combined frequency of NATs (1.5%) present or expressed in a stage-specific manner, that is, occurring only in one particular stage, was very similar to the lincRNAs (1.1%) (Fig. 1C). This suggests that maternally deposited NATs are probably more stable compared to maternally deposited lincRNAs. To assess the significance of these observations, we compared these results to the stability patterns of mRNAs during development (Supplemental Fig. S1A). In contrast to NATs and lincRNAs, lesser percentages of mRNAs were stable specifically in either pre-MZT or post-MZT (only 2% in first four stages and only 4% were stable in the six stages post-MZT). In contrast, a higher percentage of mRNAs were stable all throughout the eight stages (∼25%) compared to NATs (∼9%) and lincRNAs (∼12%).

Can these differences between NATs and lincRNAs be related to the functions of protein-coding genes they are associated with? To answer this, we compared functions of the protein-coding genes overlapping all NATs annotated in zebrafish to protein-coding genes adjacent to all lincRNAs (Fig. 1D). A Gene Ontology analysis showed that NATs significantly overlapped genes coding for DNA-binding proteins such as transcription factors (*P*-value ≤ 5.40×10^−9^). On the other hand, the top functional category for protein-coding genes adjacent to lincRNAs (within ±2 kb) was prostaglandin transmembrane transporter activity (Fig. 1D, bottom). We also saw some enrichment of transcription factor–related genes; in case of lincRNAs, however, this was much less than that seen in the case of NATs (transcription factor activity, *P*-value ≤ 7.60×10^−3^). The observation that transcription factors (TFs) were significantly overrepresented among the genes overlapping NATs might suggest that antisense transcription is a general feature of genes coding TFs. However, only 4% of all TF genes in zebrafish overlap NATs, making it unlikely that it is a general feature of TF genes (Supplemental Fig. S1B). To examine if this association of NATs and lincRNAs to the functions of associated genes is evolutionarily conserved, we carried out a similar analysis in human and mouse (Fig. 1D). In mouse, NATs were also associated with genes with DNA-binding functions; however, in humans, we did not see any significant enrichment (Fig. 1D), presumably indicating evolutionary changes in association of NATs and protein-coding genes.

Last, to understand if they are distinct in any other way, we also compared other properties of NATs and lincRNAs in zebrafish, mouse, and human. In zebrafish and mouse, lincRNAs showed a significantly higher exon count and transcript length compared to NATs (Supplemental Fig. S1C). However, in human, NATs and lincRNAs were similar in exon count and transcript length (Supplemental Fig. S1C). In all three species, NATs and lincRNAs, showed significant differences in the conservation level. To rule out the possibility that this is because of the bias introduced by differences in the genomic position of intragenic NATs compared to intergenic lincRNAs, we corrected conservation levels by taking into consideration only those NAT exons that overlap <10% of any sense-strand protein-coding exon and NAT promoters that are away from promoters of protein-coding genes and do not overlap protein-coding exons. After this correction, the conservation of NATs and lincRNAs did not show any significant difference (Supplemental Fig. S1D).

### NATs can be grouped into classes with distinct relationships to the overlapping protein-coding genes

Although a large of number of NATs are present before MZT in zebrafish, the role of NATs in gene expression regulation during early zebrafish development remains unclear. In addition, the relevance of expression changes in NATs to early embryonic development has not been fully understood. Therefore, as a first step, we sought to analyze the expression patterns of NATs during early development. Although not a proof of functionality, their coexpression patterns vis-à-vis the overlapping protein-coding genes and other genomic features can provide insights into the early events during development.

**Figure 2.**
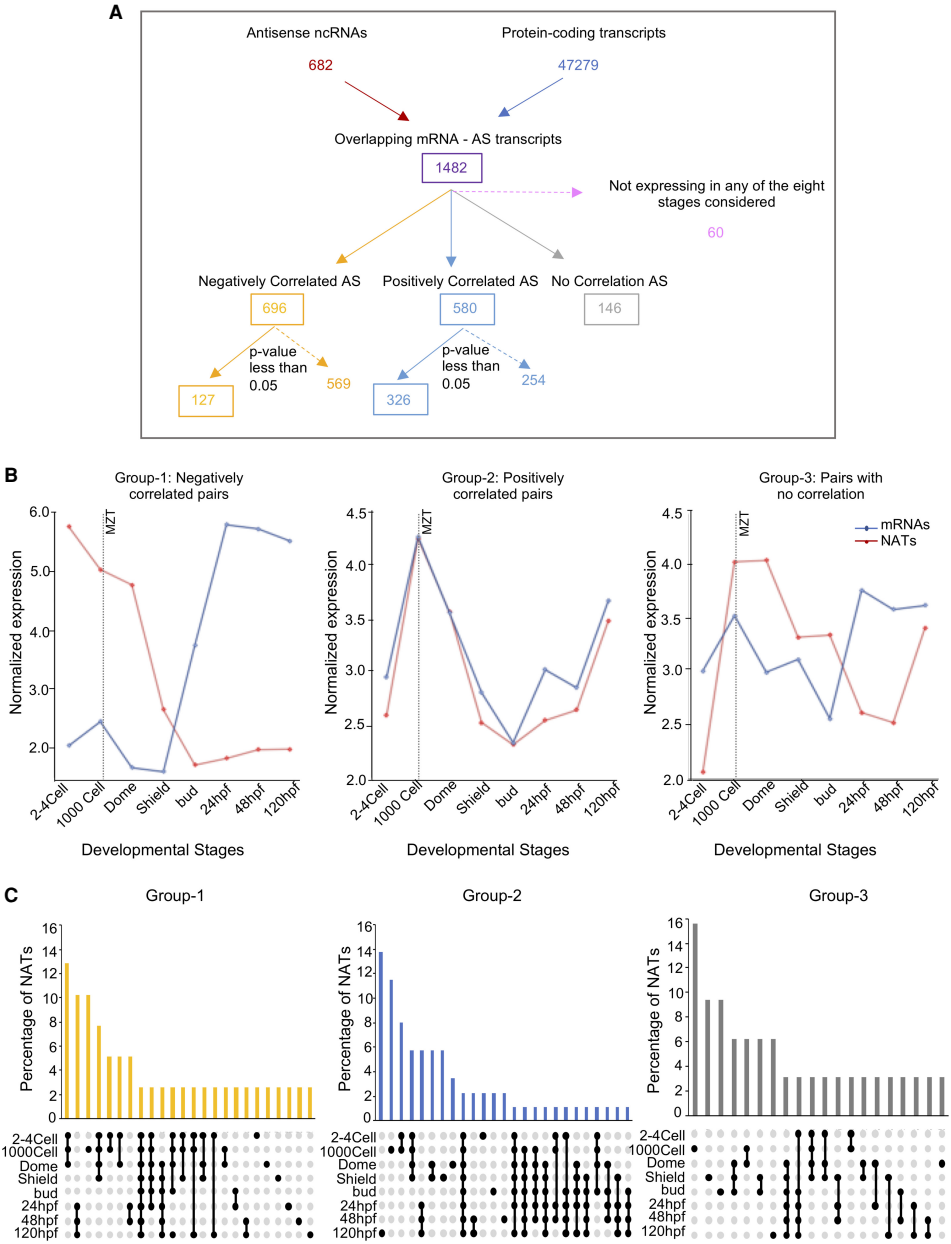
Distinct classes of NATs during zebrafish development. (*A*) Overview of the pipeline undertaken to identify and categorize the NATs based on their expression correlation with overlapping mRNAs. There are 127 anti-correlated (Group 1), 326 positively correlated (Group 2), and 146 no correlation (Group 3) NAT–mRNA pairs. (*B*) Line plots showing the average abundance of NATs and overlapping mRNAs in the three categories (negatively correlated, positively correlated, and no correlation) during development. (*C*) UpSet plots illustrating the frequency of NATs in the negatively correlated Group 1 (yellow), positively correlated Group 2 (blue), and no correlation Group 3 (gray) that are common or unique during zebrafish development. Filled circles represent the stages under consideration for the bar *above*.

For this analysis, we selected NATs that had minimum 10% overlap with the overlapping sense-strand coding transcript (Fig. 2A). Our analysis based on Ensembl annotations showed that there are 1482 such protein-coding/NAT pairs. To understand the relationship between protein-coding/NAT pairs, we carried out a correlation calculation between normalized RNA levels of each protein-coding/NAT pair across the eight developmental stages. Of the 1482 protein-coding and NAT pairs, 60 pairs did not express at all in any of the developmental stages we considered and therefore were excluded (Fig. 2A). Among remaining 1422 protein-coding/NAT pairs, 696 pairs were negatively correlated, 580 pairs were positively correlated, and the remaining 146 showed no correlation. Pairs with significant correlations (*P*-value < 0.05 and *r* > 0.71) were retained for further analysis. As a result, 127 anti-correlated (Group 1) and 326 positively correlated protein-coding/NAT pairs (Group 2) were short-listed (Fig. 2A). The 146 pairs that did not show any correlation were used as a control set (Group 3).

First, we examined if the NATs in these three groups are predominantly present in either the pre- or the post-MZT stages. To understand this, we plotted average abundance of NATs as well as mRNAs in the three groups. We found that Group 1 NATs were more abundant in the pre-MZT stages and their levels decreased in the post-MZT stages. Conversely, the protein-coding transcript levels in Group 1 increased post-MZT (Fig. 2B), thus suggesting that the NATs in the Group 1 category were predominantly maternally deposited and, like other maternal transcripts, probably degraded upon zygotic genome activation at 10 hpf (bud stage). In contrast, Group 2 and Group 3 NATs did not show any particular tendency to be abundant either pre-MZT or post-MZT (Fig. 2B). On an average, NATs and mRNAs belonging to the positively correlated Group 2 and uncorrelated Group 3 showed both maternal (0.75–10 hpf) and zygotic (24 hpf onward) presence. Interrogation of individual RNA levels in Group 1 and Group 2 confirmed that the average expression patterns reflect correlations between individual protein-coding/NAT pairs (Fig. 2B; Supplemental Figs. S2, S3).

These results were comparable to that obtained by another frequently used RNA-seq analysis software, STAR (Dobin and Gingeras 2015). A comparison showed that, independent of the alignment protocols, ∼80% of NAT/protein-coding pairs showed similar correlation and are classified in the same groups as our original pipeline (Supplemental Fig. S4A). In addition, the relationship between expression patterns of NATs and their overlapping protein-coding partners in the three groups was very similar using the two alignment protocols (Fig. 2B; Supplemental Fig. S4B). To rule out the possibility that coexpression relationships between NAT/protein-coding pairs is a mere consequence of expression changes that occur during MZT and is not affected if the NATs were to be randomly paired with nonoverlapping mRNAs, we paired NATs with neighboring nonoverlapping protein-coding transcripts as well as with protein-coding transcripts expressed elsewhere in the zebrafish genome. In both the scenarios, ∼64%–80% of NATs showed no correlation, and ∼8%–18% showed the opposite relationship with randomly selected mRNAs compared to the overlapping protein-coding transcript partner (Supplemental Fig. S4C,D). This suggests that the correlations in the RNA levels between NATs and their overlapping protein-coding partners is not random and is independent of genome-wide gene expression changes linked to maternal-to-zygotic transition.

If Group 1 NATs were maternally deposited, then like other maternal RNAs, this group of NATs should show distinct stability pre-MZT and post-MZT. Therefore, using UpSet plots, we also analyzed if NATs in the three groups showed differences in the stability during MZT (Fig. 2C; Supplemental Table S2). This analysis showed that a large number of the NATs in the Group 1 category were present in the early stages (2–4 cell, 1000 cell, and dome; 13%; χ^2^ *P*-value < 0.005). The percentage of Group 1 NATs present only in a single stage was very low, suggesting that they are not highly stage specific. In contrast, the highest percentage for Group 2 NATs was observed in single stages (14% for 120 hpf and 11% for 1000 cell). Moreover, the stability pattern of Group 2 was very comparable to the control Group 3 NATs. Thus, suggesting that the Group 1 NATs are more stable and are present through multiple stages spanning MZT as compared to the Group 2 NATs and the Group 3 NATs. These results show that during early vertebrate development two very different classes of NATs are present, and they show distinct relationship to their overlapping protein-coding genes.

### NATs in different groups are distinct in their organization vis-à-vis the overlapping genes

Examples supporting positive as well as negative regulation of protein-coding genes by NATs are described in the literature (Wang and Chang 2011; Wight and Werner 2013). In some cases, NATs are shown to have no effect on the function of the overlapping protein-coding partner or might instead have an effect on a gene at distant loci (Wight and Werner 2013). This is reflected in our analysis, which shows that antisense RNA-protein pairs can be divided into either negative (Group 1), positive (Group 2), or no-correlation groups (Group 3). Besides displaying different coexpression relationships, NATs are also known to be organized in a different manner vis-à-vis the overlapping gene. Well-studied NATs are shown to be transcribed near the 5′-end (*HOTTIP*), near the 3′-end (*HOTAIRM1*), or from the gene body of the sense-strand gene (*BACE1-AS, Kcnq1ot1*). Understanding how different categories of NATs are organized in relation to overlapping protein-coding genes would help in postulating reasons behind positive, negative, and no-correlation patterns between NATs/protein-coding pairs as well as gaining insights into functional mechanisms of NATs. However, the link between coexpression patterns between NAT/protein-coding gene pairs and their genomic positioning relative to each other has not been clearly understood.

**Figure 3.**
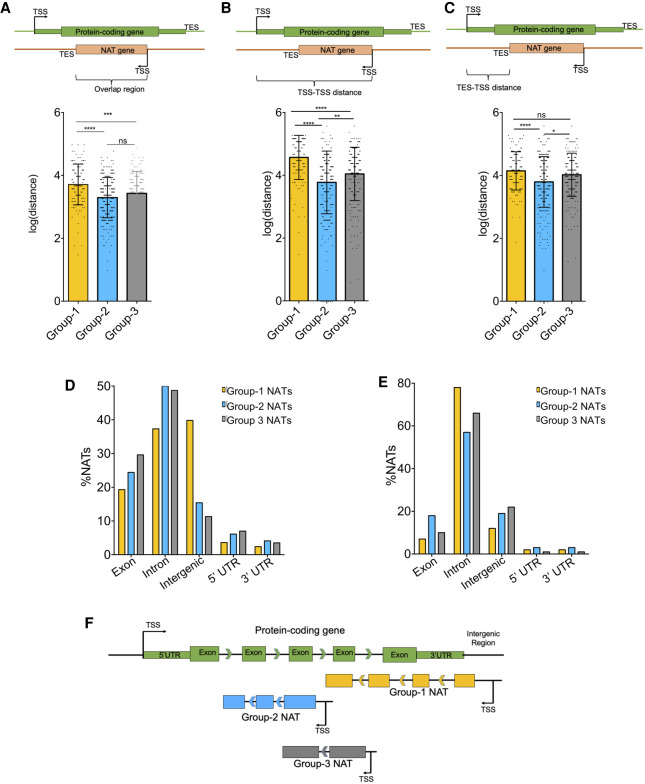
The NATs in three categories are differently transcribed vis-à-vis overlapping protein-coding genes. (*A*) A bar chart showing the overlap region between NATs and overlapping mRNAs in Group 1 (yellow), Group 2 (blue), and Group 3 (gray). (*B*) A bar chart showing the distance between the TSS of NATs and the TSS of overlapping mRNAs (log of distance in bp) in Group 1 (yellow), Group 2 (blue), and Group 3 (gray). (*C*) A bar chart showing the distance between the TES of NATs and the TSS of overlapping mRNA (log) in the Group 1 (yellow), Group 2 (blue), and Group 3 (gray). For *A*, *B*, and *C*, the schematic *above* each figure represents how the values were calculated. The bar values represent the mean ± standard deviation. The scatter shows individual values. The significance values are as follows: (*) *P* < 0.05; (**) *P* < 0.005; (***) *P* < 0.001; (****) *P* < 0.0001; (ns) nonsignificant (unpaired, two-tailed *t*-test). (*D*) A bar plot of the percentage of NAT TSSs overlapping different genomic features (exon, intron, intergenic region, 5′ UTR, and 3′ UTR) on the opposite strand. (*E*) A bar plot of the percentage of NAT TESs overlapping different genomic features (exon, intron, intergenic region, 5′ UTR, and 3′ UTR) on the opposite strand. (*F*) Schematic diagram representing how three different groups of NATs are distributed and localized in the genome with respect to the overlapping protein-coding genes.

We first calculated the extent of the overlap between NAT and overlapping protein-coding gene pairs in the three categories. We defined NAT/protein-coding overlap as the percent length of the protein-coding gene that is completely covered by the NAT in the antisense direction (Fig. 3A; Supplemental Fig. S5A). NAT/protein-coding transcript pairs in Group 1 showed significantly greater overlap (average = 13,003 bp, *P*-value < 0.01) as compared to the pairs in Group 2 (average = 4548 bp) and in the no-correlation group (average = 6722bp). To further understand the nature of NAT/protein-coding transcript overlap, we also calculated TSS–TSS distances, that is, genomic distances between the transcription start sites (TSSs) of NAT and protein-coding genes in a pair (Fig. 3B). TSS–TSS distance distribution in the three groups of NATs was distinct from one another (Fig. 3B; Supplemental Fig. S5B). The average TSS–TSS distance in Group 1 NAT and overlapping protein-coding TSSs [log(distance) = 4.6] was much greater than that in Group 2 [log(distance) = 3.6] and Group 3 [log(distance) = 4.0]. However, when we calculated TES–TSS distances, that is, genomic distances between transcription end sites (TES) of NATs to the TSSs of protein-coding genes in the pair, we found that TES–TSS distances in Group 1 [average log(distance) = 4.1] and Group 2 [average log(distance) = 3.7] were significantly different (*P*-value < 0.01) (Fig. 3C; Supplemental Fig. S5C). However, Group 1 was similar to the control Group 3 [log(distance) = 4.0] in this aspect.

We further interrogated whether NATs in different groups also start and end distinctly vis-à-vis genomic features—namely, exons, introns, intergenic regions, 5′ UTRs, and 3′ UTRs of protein-coding genes on the opposite strand (Fig. 3D,E). The majority of NATs (∼40%) in Group 1 started in intergenic regions in contrast to the Group 2 and Group 3 NATs, which mainly started within the introns (50% and 48.7%, respectively) of the protein-coding gene on the opposite strand (Fig. 3D). On the contrary, the TESs of a large majority of NATs in all the three categories were in the introns of protein-coding genes on the sense strand (Fig. 3E).

This shows that Group 1 NATs start much further away from protein-coding TSSs compared to Group 2 NATs. However, the Group 2 NATs end nearer to the protein-coding TSSs compared to Group 1 and Group 3 NATs. This might suggest that the average length of NATs in Group 1 might be more than other groups. However, transcript lengths in Group 1 NATs were only marginally greater than Group 2 and Group 3 (Supplemental Fig. S5D). The number of exons in Group 1 NATs was significantly higher than other two groups (Supplemental Fig. S5E).

These observations can be summarized in a model showing that NATs in Group 1 start in intergenic regions and span a large percentage of the length of overlapping protein-coding gene (Fig. 3F). Group 2 and Group 3 NATs, conversely, start in introns of the overlapping protein partner. Group 2 NATs end in introns nearer to the TSS of overlapping protein-coding gene (Fig. 3F).

### Group 1 NATs overlap developmental genes, and Group 2 NATs overlap housekeeping genes

**Figure 4.**
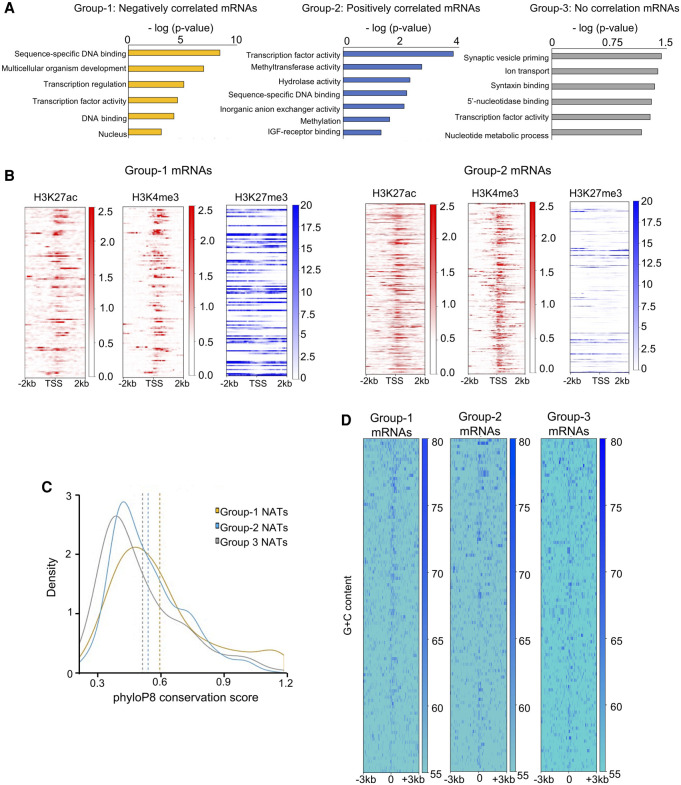
Negatively correlated NATs overlap protein-coding genes involved in vertebrate development. (*A*) Bar plots depicting the Gene Ontology terms associated with mRNAs in the three categories of NAT protein-coding pairs. In each case, the *x*-axis displays −log(*P*-value) for Gene Ontology terms that are shown on the *y*-axis. (*B*) Heatmaps displaying the distribution of ChIP-seq reads for H3K27ac (red), H3K4me3 (red), and H3K27me3 (blue) histone modifications across the TSS of mRNAs overlapping Group 1 and Group 2 NATs. (*C*) Density plots showing the conservation score (phyloP8) at the promoters of mRNAs. Group 1 mRNAs are more conserved in comparison to the other two groups. (*D*) Heatmaps displaying G + C content at the promoters of mRNAs in the three categories.

Given the distinct expression patterns of NATs and protein-coding transcripts in the three groups (Fig. 2B), a pertinent question would be whether the protein-coding genes in these groups are distinct in their biological and cellular functions. To address this question, a Gene Ontology (GO) analysis was carried out on the protein-coding transcripts in the three groups. We found that the genes in the three groups were enriched in distinct molecular functions (Fig. 4A). The transcripts belonging to Group 1 were highly enriched in sequence-specific DNA-binding proteins (*P*-value < 3.70×10^−9^), developmental genes (*P*-value 1.40×10^−7^), and proteins involved in transcription regulation (*P*-value < 8.90×10^−6^). The genes involved in transcription factor activity were also enriched in Group 2 (*P*-value < 1.60×10^−4^), however, less significantly than the Group 1 protein-coding genes (*P*-value < 3.70×10^−9^). In addition, Group 2 genes also showed enrichment of housekeeping functions related to metabolism and signaling processes. In contrast, the transcripts in the noncorrelated Group 3 did not show significant enrichment of any particular category.

To confirm the association of Group 1 NATs with developmental genes, we sought to investigate if these genes also display other characteristics of developmental genes. It is well-documented that during early stages of development, genes involved in developmental pathways are repressed by polycomb group of proteins. Polycomb Repressive Complex-2 catalyzes trimethylation of the lysine 27 on the histone H3 (H3K27me3) at the promoters of developmental genes (Aloia et al. 2013; Deevy and Bracken 2019). Given the enrichment of developmental genes in Group 1 (Fig. 4A), we expected that the promoters of protein-coding genes in this group would show histone mark patterns, such as enrichment of H3K27me3, that are normally seen at developmental genes. We mined genome-wide chromatin immunoprecipitation data (ChIP-seq) for three different histone modification marks in the dome stage of zebrafish development. In addition to H3K27me3 modification, we also analyzed H3K4me3 (Histone 3 lysine 4 trimethylation) and H3K27ac (Histone 3 lysine 27 acetylation) marks. H3K27me3 mark is largely enriched at transcriptionally repressed genes, whereas H3K4me3 and H3K27ac marks are usually enriched at transcriptionally active genes (Vastenhouw et al. 2010; Black et al. 2012; Zhang et al. 2014). We checked the enrichment of these marks at the TSSs of protein-coding transcripts in Group 1, Group 2, and the control Group 3. We observed distinct patterns of histone modifications across TSSs overlapping protein-coding genes in the different groups. As predicted, protein-coding genes in Group 1 showed higher enrichment of repressive H3K27me3 mark (Fig. 4B; Supplemental Table S3). H3K27ac and H3K4me3 marks did not show as significant enrichment at the TSS of protein-coding genes in Group 1 as in Group 2. The relative lack of H3K4me3 and H3K27ac marks and the enrichment of H3K27me3 mark suggests that Group 1 protein-coding partners are not transcribed. In contrast, the histone modification marks at the Group 2 genes were distinct. The protein-coding genes in Group 2 showed much lower enrichment for the H3K27me3 repressive mark (Fig. 4B; Supplemental Table S3). However, unlike Group 1, there was a significant enrichment of H3K27ac and H3K4me3 marks across their TSSs (Fig. 4B, right panels). The histone modification patterns at Group 2 promoters are very similar to that observed at average protein-coding genes (Supplemental Fig. S6A), again reflecting that Group 2 probably represents genes with housekeeping functions as shown in our analysis of functional categories (Fig. 4A). On the other hand, Group 3 protein-coding genes did not show any distinctive enrichment for any histone modifications (Supplemental Fig. S6B), indicating they do not display a particular pattern of expression.

Evolutionary conservation of genes or genomic elements is often used as a proxy for their functional importance. Developmental genes are often more conserved than average genes. We assessed evolutionary conservation of the gene promoters, 3′ UTR, and 5′ UTR in the two categories (Fig. 4C; Supplemental Fig. S6C,D). As would be expected for developmental genes, Group 1 showed higher conservation score at the promoters (*t*-test; *P*-value < 0.05) compared to Group 2 and the control Group 3 (Fig. 4C). Because the polycomb group of genes have GC-rich promoters, we further analyzed the GC content at the promoters of different groups. Our analysis suggests that the Group 1 protein-coding genes have promoters with higher G + C content compared to Group 2 genes and control Group 3 (Fig. 4D). The G + C content at 5′ UTRs and 3′ UTRs, however, was not different between the protein-coding genes in the three classes (Supplemental Fig. S6E,F).

These observations support that the protein-coding genes in Group 1 are polycomb-targeted developmental genes. Conversely, Group 2 and noncorrelated groups were likely housekeeping genes, suggesting a different mechanism of regulation.

### NAT expression is regulated in a group-specific manner

**Figure 5.**
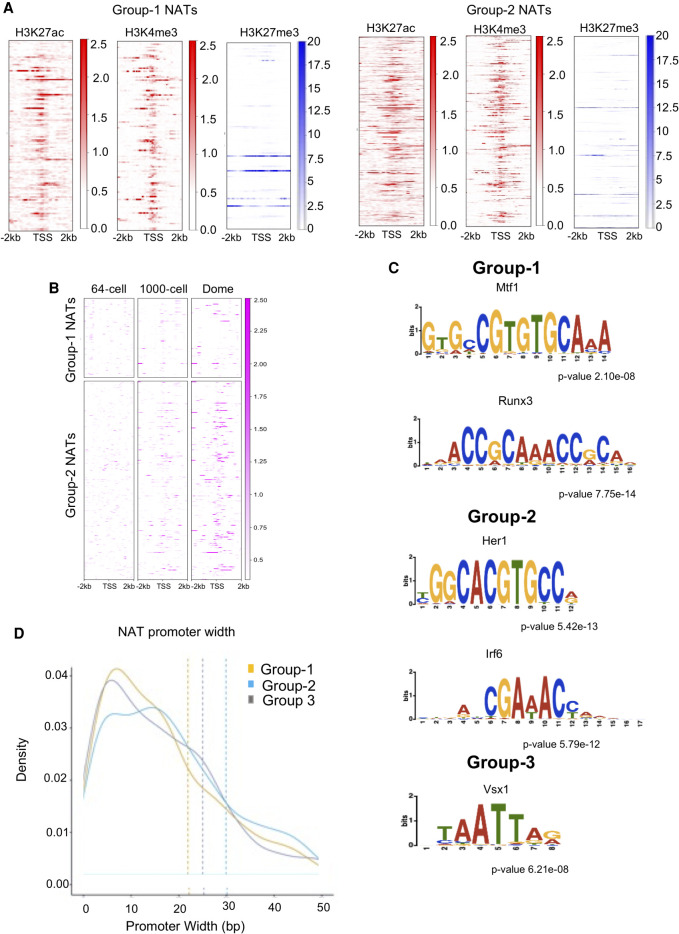
Analysis of transcription regulation of NATs in the three different categories. (*A*) Heatmaps displaying the distribution of ChIP-seq reads for H3K27ac (red), H3K4me3 (red), and H3K27me3 (blue) histone modifications across the TSS of Group 1 and Group 2 NATs. (*B*) Heatmaps of the distribution of ATAC-seq reads displaying the open chromatin at the TSS of Group 1 and Group 2 NATs. (*C*) The different motifs associated within the promoters of Group 1, Group 2, and Group 3 NATs. (*D*) Promoter width (*x*-axis) distribution of the NATs in the three groups.

Because we observed differences in protein-coding genes in different groups, we also sought to understand the differences in the transcription regulation of NATs. First, we plotted histone modifications and chromatin accessibility at the TSS of a different category of NATs. As observed in the case of protein-coding genes, distinct patterns of histone modifications were observed across the TSS of NATs in the different groups (Fig. 5A; Supplemental Table S3). Unlike Group 1 protein-coding genes, NATs in Group 1 showed little or no enrichment of the H3K27me3 mark around the TSS, indicating that they were probably not repressed by polycomb machinery. In addition, H3K27ac and H3K4me3 marks were less enriched at Group 1 NAT TSSs compared to Group 2. This is in accordance with our prediction that Group 1 NATs are maternally deposited rather than zygotically transcribed. This was also reflected in open chromatin status as measured by ATAC-seq method (Fig. 5B), which showed that the Group 1 NAT promoters do not show presence of open chromatin supporting absence of transcription. In contrast to Group 1, Group 2 NATs showed enrichment of H3K27ac and H3K4me3 marks across their TSSs (Fig. 5A, right panels). This was supported by ATAC-seq data showing more open chromatin than Group 1. Conversely, Group 3 NATs did not show enrichment for any histone modifications (Supplemental Fig. S6B, top panels), indicating they do not display a particular pattern of expression.

The distinct expression patterns of NATs and functional roles of overlapping protein-coding genes in the three categories point to differences in NAT transcription regulation. To further dissect the nature of transcription regulation of NATs, we analyzed the sequence motifs associated with the promoters of NATs in these three categories. As expected, NATs in the three categories showed enrichment of distinct transcription factor (TF) motifs (Fig. 5C). We observed that promoters of Group 1 NATs showed enrichment for TF motifs such as Mtf1 and Runx3, which are known to be essential for normal vertebrate development (Burns et al. 2002; Oates and Ho 2002; Hogstrand et al. 2008). The promoters of Group 2 category NATs were enriched instead in Her1 and Irf6 transcription factor motifs that are also involved in zebrafish development (Fig. 5C).

Another indication of transcription regulation is provided by the promoter width (Haberle et al. 2014). The existence of a sharp TSS in the case of maternal transcripts and a broad TSS in the case of zygotic transcripts has been reported (Haberle et al. 2014, 2015). The promoter width can be effectively calculated by mapping the TSS using Cap Analysis Gene Expression (CAGE) analysis. Using CAGE, changes in TSSs and its relative usage can be measured at single-nucleotide resolution (Kodzius et al. 2006). CAGE gives us the information for the start sites of capped RNAs, which in turn can be used as an indicator of promoter organization. The width of TSSs can be calculated based on the mapped CAGE tags (Haberle et al. 2015). For this analysis, we deep sequenced the CAGE tags from six stages of early zebrafish development and calculated the promoter width (Fig. 5D). In this analysis, Group 2 NATs appeared to have much broader promoters compared to promoters of Group 1 NATs and the control Group 3. This observation further supports the maternal nature of Group 1 NATs and the housekeeping nature of Group 2 NATs.

### NATs show differences in cellular localization

Subcellular localization of RNAs can give an indication regarding their functional mechanism (Cabili et al. 2015). Studies on yeast (Long et al. 1997) and flies (Johnstone and Lasko 2001) have indicated very specific subcellular localization of mRNAs to be important in yeast and fly development. It can be envisioned that the specific subcellular localization of NATs during zebrafish embryogenesis might also be linked to their cellular function. For example, lncRNAs involved in regulation of chromatin-modification and transcription are localized in the nucleus and lncRNAs involved in post-transcriptional regulation are localized in cytoplasm (Cabili et al. 2015). Keeping this in mind and to get an idea regarding the functional mechanisms of Group 1 and Group 2 NATs, we sought to identify the subcellular localization of a different category of NATs during early stages of zebrafish embryos. To achieve this, we carried out deep sequencing of RNAs from cytoplasmic and nuclear fractions collected from different stages of development. An RNA was categorized as more enriched in the nuclear fraction if its abundance was 1.5-fold or more when compared to the cytosolic fraction. Similarly, an RNA was considered more enriched in the cytosol if its abundance was 1.5-fold or more in the cytoplasm than that in the nuclear fraction. RNAs with abundance between 0 and 1.5-fold were considered to be equally present in both cellular fractions.

To verify if our protocol correctly identifies subcellular localization of RNAs, we first analyzed the localization of mRNAs. The mRNAs were identified as predominantly equally present in both cellular fractions or cytosolic showing that our protocol for identifying subcellular localization of RNAs worked well (Supplemental Fig. S7A).

**Figure 6.**
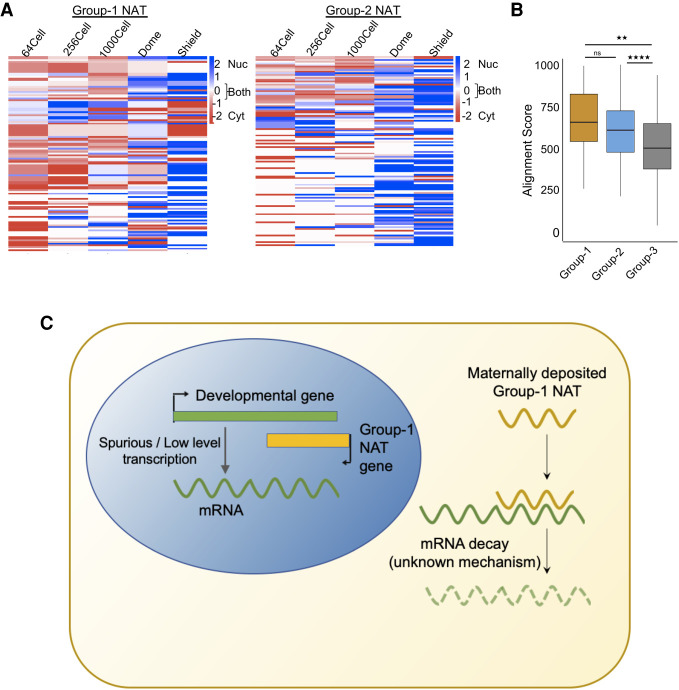
Localization of NATs during zebrafish development. (*A*) Heatmaps showing the localization of Group 1 and Group 2 NATs during zebrafish development. The scales are from −2 (red, cytosolic) to 2 (blue, nuclear) and 0 (white, both). (*B*) A box plot of the alignment score between NATs and overlapping mRNA sequence in Group 1 (yellow), Group 2 (blue), and Group 3 (gray). Each box shows the median value (as horizontal lines) and extends from the 25th to 75th percentile values for each group. The outliers are shown as dots. The significance values are as follows: (**) *P* < 0.005; (****) *P* < 0.0001; (ns) nonsignificant (unpaired, two-tailed *t*-test). (*C*) A schematic model showing probable mechanism of Group 1 NATs in down-regulating developmental genes.

We then analyzed cellular localization of NATs in the three groups. We ranked NATs based on their abundance and then calculated the log_2_ ratio to determine their localization. Group 1 NATs were more enriched in the cytosolic fraction during pre-MZT stages further supporting maternal deposition and become more enriched in the nuclear fraction in the shield stage presumably indicating the beginning of zygotic transcription (Fig. 6A). The majority of Group 2 NATs were equally enriched in both the fractions at the 64-cell stage; however, after the 64-cell stage, they were more enriched in the nucleus (Fig. 6A). The control group NATs were in the cytoplasm at the 64-cell stage but did not show any particular enrichment after the 64-cell stage (Supplemental Fig. S7B).

Previous studies have indicated that cytosolic NATs that down-regulate the overlapping protein-coding genes act by forming an RNA:RNA hybrid leading to post-transcriptional changes in mRNA stability (Villegas and Zaphiropoulos 2015). Given the cytosolic localization of Group 1 NATs, a post-transcriptional mechanism involving RNA–RNA hybridization can be envisioned. Given the negative correlation of Group 1 NATs with the levels of their overlapping mRNAs, it can be speculated that they form RNA:RNA hybrids with the sense-strand mRNA partners and decrease their stability. To check this possibility, we assessed the sequence complementarity between NAT transcripts and corresponding mRNAs in the pairs in the three groups (Fig. 6B). In this analysis, Group 1 NATs showed higher complementarity as reflected in the alignment score compared to the control Group 3 (*P*-value < 0.0001) (Fig. 6B). The cytosolic localization and higher complementarity indicate a post-transcriptional mechanism for Group 1 NATs for regulation of mRNA expression. We speculate that Group 1 NATs, possibly through RNA:RNA interaction, help to repress developmental gene expression (Fig. 6C).

## Discussion

Antisense transcription is a common feature among all types of organisms ranging from bacteria to mammals with up to 40% of the human transcriptome being predicted to show evidence of antisense transcription (Chen et al. 2004; Katayama et al. 2005; Zhang et al. 2006; He et al. 2008; Balbin et al. 2015). A large proportion of protein-coding mRNAs are part of sense-antisense transcript pairs in which the antisense transcript is a lncRNA (Chen et al. 2004; Katayama et al. 2005; Engström et al. 2006; Derrien et al. 2012; Hon et al. 2017; Zucchelli et al. 2019). In many cases, NATs regulate the overlapping protein-coding gene on the sense strand (Villegas and Zaphiropoulos 2015). Despite the widespread nature of antisense transcription, its functional significance remains poorly characterized. The functional relationship of NATs to the overlapping sense-strand protein-coding genes still remains confusing. One of the reasons behind this is that NATs display positive as well as negative correlation to the expression of their sense-strand partners (Faghihi and Wahlestedt 2009; Pelechano and Steinmetz 2013; Wight and Werner 2013; Khorkova et al. 2014; Rosikiewicz and Makalowska 2016). In addition, a number of NATs do not affect the expression of overlapping genes. In some cases, the antisense transcription can result merely as a consequence of opening of chromatin owing to transcription of the sense-strand gene or as a by-product of enhancer activity (Struhl 2007; Onodera et al. 2012). In other instances, they are shown to regulate protein-coding genes at other nonoverlapping loci (Roberts and Morris 2013; Rosikiewicz and Makalowska 2016). As a result, it has been difficult to propose a unifying mechanism behind their function.

Here, we focused on analyzing the relationship between NATs and their overlapping protein-coding genes during zebrafish development. Previous studies took an approach in which they first divided NATs according to their genomic location vis-à-vis their overlapping protein-coding gene pair and then analyzed the coexpression pattern between NAT/protein-coding pairs (Balbin et al. 2015). Instead, we focused on first grouping NATs according to the coexpression patterns of NAT/protein-coding pairs across eight stages of zebrafish development and then analyzing their characteristics.

Different sets of NATs/protein-coding pairs show distinctive characteristics. The Group 1 protein-coding genes are mainly developmental genes, whereas Group 2 protein-coding genes show enrichment for housekeeping functions (Fig. 4). Conversely, Group 3 did not show enrichment for any particular functional pathways. Group 1 and Group 2 NATs were also distinctive in terms of positioning of transcription start and end sites as well as promoter structure, indicating that their expression is regulated in a distinct manner (Figs. 3, 5). This is reflected in their expression pattern during development, indicating Group 1 NATs are maternally deposited whereas Group 2 RNAs seem to be transcribed from zygotic genome (Fig. 2). Our RNA-seq data show that Group 1 RNAs are cytosolic, which is also typical of maternal RNAs (Fig. 6). Group 2 RNAs on the other hand show more nuclear presence, possibly because they are transcribed from the zygotic genome (Fig. 6).

Previous studies on NATs have shown they can regulate either transcription or post-transcriptional processing (Faghihi and Wahlestedt 2009; Pelechano and Steinmetz 2013; Wight and Werner 2013; Khorkova et al. 2014; Rosikiewicz and Makalowska 2016). Given the predominantly cytosolic nature of Group 1 RNAs, it is unlikely that they are involved in transcriptional regulation. Previous publications have indicated that antisense transcription from intragenic enhancers can have an attenuating effect on transcription of the overlapping protein-coding genes (Cinghu et al. 2017). However, unlike Group 1 NATs, these intragenic enhancers were predominantly found to be enriched in chromatin fraction (Cinghu et al. 2017). Besides in our analysis (Fig. 5), neither Group 1 nor Group 2 NATs display classic enhancer signature, enrichment of H3K27ac mark, and lack of H3K4me3 mark, indicating that the majority of Group 1 and Group 2 NATs are not by-products of enhancer activity.

However, based on their cytosolic localization and opposite expression pattern compared to overlapping developmental mRNAs, we can speculate that Group 1 NATs function by changing the stability of developmental mRNAs during early stages of zebrafish development (Fig. 6C). They might be involved in decreasing mRNA stability through formation of RNA:RNA hybrids as reported for other cytosolic NATs. This is corroborated by the higher level of complementarity in the Group 1 antisense RNA–mRNA pairs, which alludes to a possibility of hybridization between antisense RNA and mRNA. Examples show that formation of RNA duplex between lncRNAs and mRNAs can attract mRNA-degradation machinery as seen in the case of STAU1-mediated mRNA degradation (Gong and Maquat 2011b). In addition, lncRNAs are also implicated in generating endogenous siRNAs, which can lead to mRNA degradation (Wilusz et al. 2009; Gong and Maquat 2011a). One of the questions that is highlighted from our analysis is the reason behind the higher percentage of Group 1 NATs among maternally deposited RNAs. It is possible that they do not have any significant role in regulating the developmental genes. However, their expression specifically from developmental gene loci and their distinct features when compared to the other two groups of NATs point to their significance. It can be speculated that Group 1 NATs are needed to curtail unwarranted expression of developmental genes during early stages before MZT, which can be detrimental to normal development. However, functional characterization and further experimental validation is needed to confirm these observations.

In addition to functional differences between Group 1 and Group 2 mRNAs, we also see differences in the genomic location of Group 1 and Group 2 NATs vis-à-vis overlapping mRNAs. A majority of Group 1 NATs start in an intergenic region, away from the TSS of their overlapping protein-coding partner and generally display larger overlap with the sense gene. In contrast, Group 2 NATs start much closer to the sense gene TSS, and they appear to be in a head-to-head or an embedded configuration with respect to the sense protein-coding gene. We speculate that specific configuration plays a role in deciding the relationship between NATs and their overlapping protein-coding genes. This kind of relationship has been reported in previous studies and individual examples that showed that head-to-head configuration is associated with positive coexpression patterns between antisense/protein-coding pairs (Balbin et al. 2015). Distinct features observed in negatively correlated Group 1 and positively correlated Group 2 can be useful in predicting NAT protein-coding coexpression in the future. Further experimental studies are however needed to understand the exact nature of the relationship between NATs and their protein-coding pair and also to verify if these observations are broadly applicable to the development of other vertebrates.

Last, for this study we have generated a large transcriptomics data set (RNA-seq and CAGE-seq), which can provide information regarding RNA enrichment in different cellular compartments. In addition to NATs, these data will be useful to assess roles of other ncRNAs such as enhancer RNAs and circular RNAs during development.

## Methods

### Analysis of RNA-sequencing data

Expression levels of NATs and mRNA was calculated using RNA-sequencing data for eight zebrafish developmental stages (Pauli et al. 2012). FASTQ files corresponding to raw RNA-sequencing reads for eight zebrafish developmental stages were downloaded from the NCBI Gene Expression Omnibus (GEO; https://www.ncbi.nlm.nih.gov/geo/) under accession number GSE32900. They were quality checked and trimmed using FastQC (http://www.bioinformatics.babraham.ac.uk/projects/fastqc/) and Trimmomatic v0.27, respectively (Bolger et al. 2014). Trimmed and quality filtered sequencing reads were then mapped back to the Zv9 or danRer7 genome assembly of zebrafish using two different alignment pipelines. In case of the first pipeline, the reads were mapped to the genome using TopHat suit (Kim et al. 2013), and then the aligned reads were used for transcript assembly and expression levels using Cufflinks v2.2.1 (Trapnell et al. 2012). The expression levels were measured by Cufflinks as fragments per kilobase per million (FPKM) reads for each transcript. For the second pipeline, the raw sequencing reads were also mapped to the Zv9 genome assembly of zebrafish using another alignment program STAR v2.6.0a (Dobin and Gingeras 2015), and the generated alignment files were used to assemble transcripts using the program StringTie (Pertea et al. 2015). The StringTie program was run using the -B and -b options. The output of StringTie was used for differential expression analysis using the program Ballgown (Pertea et al. 2016). The transcript and gene levels we calculated as transcripts per million (TPM) for different stages of development. The outputs of Cufflinks and StringTie files were used for all our downstream analysis. The stage-specific abundance of NATs and lincRNAs was plotted and visualized using UpSet plots in R (Lex et al. 2014). For visualization purposes, strand-specific expression tracks were generated using “genomecov” command in the BEDTools package (Quinlan and Hall 2010).

### Comparison of lincRNAs and NAT characteristics

All the analysis of lincRNAs and NATs was carried out on Ensembl annotations for Zv9, mm10, and hg19 assemblies for zebrafish, mouse, and human genomes, respectively. Because this analysis relies on average and comparative characteristics, the genome versions (e.g., GRCh38 or Zv10) would not significantly affect the conclusions. LincRNAs and NATs were considered present if their expression level is ≥1 FPKM, which was calculated as described above. The transcript lengths and exon counts were calculated based on Ensembl annotations.

### Identification of protein-coding and NAT pairs

We first paired NATs with overlapping protein-coding mRNA transcripts. For this analysis, we considered 47,279 protein-coding transcripts and 682 NATs that are annotated by Ensembl for zebrafish genome version Zv9 (Zerbino et al. 2018; Yates et al. 2020). Using the genomic coordinates, we identified all the pairs in which the antisense gene overlapped at least 10% of the protein-coding gene. Based on this criterion, we identified 1482 NAT/protein-coding pairs. The percent length of mRNA transcript that was overlapped with antisense RNA was calculated using BEDTools suite's “Intersect” command using the -S option (Quinlan and Hall 2010).

### Functional analysis of NAT–mRNA pairs

The expression levels of protein-coding and NATs calculated using Cufflinks and StringTie were first normalized with respect to the maximum level of each RNA among the eight stages. The normalized values were then used to compute linear correlation between each pair. The pairs were categorized based on whether the pairs correlated negatively (Group 1), positively (Group 2), or did not show any correlation at all (Group 3). Only pairs with significant correlation were retained (*P*-value < 0.05, *r* ≥ 0.70 for sample size *N* = 8). The analysis of functional annotations was carried out using Database for Annotation, Visualization and Integrated Discovery (DAVID 6.8) software for functional annotation of these transcripts (Huang da et al. 2009a,b). For this analysis, categories related to biological process, molecular function, and cellular components were considered, and a background of all zebrafish genes was used. R package, ggplot2 (Wickham 2016), was used for plotting all of our violin and box plots. The geom_histogram() function in ggplot2 was used to plot the histograms for showing the overlap region and the distance between the TSS of protein-coding genes and the antisense TES. Heatmaps related to NATs and their overlapping protein-coding genes were also plotted using R (R Core Team 2019) and deepTools2 (Ramírez et al. 2016). Base-specific conservation scores (Vertebrate Cons) corresponding to each NAT and mRNA sequence were downloaded from UCSC Genome Browser (Karolchik et al. 2004). The conservation values for NATs were corrected by only considering promoters and exons that overlap <10% with the protein-coding exons on sense strand.

The G + C content track for zebrafish (danRer7.gc5Base.wig) from UCSC Genome Browser was used to calculate the G + C content at the promoters (Haeussler et al. 2019). We also used EMBOSS geecee analysis to calculate the frequency of G and C nucleotide in the sequences (Rice et al. 2000). The EMBOSS Needle analysis was run with the default options for pairwise sequence alignment of NATs and mRNA sequence. The sequence for mRNA and NATs was downloaded from the UCSC Genome Browser.

### ChIP-seq and ATAC-seq analysis

The raw chromatin immunoprecipitation followed by sequencing (ChIP-seq) data for different histone modifications H3K27me3 (DCD003227SQ), H3K4me3 (DCD003231SQ), and H3K27ac (DCD003287SQ) corresponding to the Dome (4 hpf) stage of zebrafish development (Vastenhouw et al. 2010; Bogdanovic et al. 2012; Zhang et al. 2014) were obtained from the DANIO-CODE repository at https://danio-code.zfin.org. The ChIP-sequencing reads were downloaded in FASTQ format and mapped to the Zv9 version of zebrafish genome using Bowtie 2 (Langmead and Salzberg 2012). The mapped reads were analyzed using ChIP-seq analysis software HOMER to create tag directories and to annotate peaks using the makeTagDirectory and findPeaks tools (-style factor and -o auto options). The processed ChIP-sequencing data was used to plot heatmaps with the help of deepTools2 (Ramírez et al. 2016) and to visualize the enrichment of different histone modifications within ±2 kb distance of transcription start sites of protein-coding transcripts as well as NATs in the three different groups of NAT/protein-coding pairs. The statistical significance of histone modification enrichments around transcription start sites of different NAT groups was calculated using BEDTools Fisher program (Quinlan and Hall 2010).

The FASTQ files for ATAC-seq data for different stages (DCD003157SQ, DCD003146SQ and DCD003127SQ) of zebrafish development (Vastenhouw et al. 2010; Bogdanovic et al. 2012; Zhang et al. 2014) were downloaded from the DANIO-CODE repository at https://danio-code.zfin.org. The raw reads were mapped to the Zv9 genome using Bowtie 2 (Langmead and Salzberg 2012). The BAM files generated were used to get bigWig files from deepTools using BAMCoverage (–normalizeUsing BPM option). The bigWig files were mapped to visualize open chromatin within ±4 kb distance of transcription start sites for both NATs and overlapping mRNAs.

### Nuclear and cytosolic fractionation of zebrafish embryos in different stages of development

Wild-type male and female zebrafish (AB-strain) were set up in breeding tanks overnight; on the next day, to ascertain their synchronization, the fertilized eggs were collected with minimum delay (<10 min). About 100–500 (depending on the stage) embryos were collected for each developmental stage (32 cells, 64 cells, 256 cells, 512 cells, high, shield). The embryos were treated with Pronase (Sigma-Aldrich) to remove the chorion. One milliliter of Pronase working solution (1 mg/mL) was added to 2–3 mL of fish water containing embryos. Dechorionated embryos were then transferred to a 1.5-mL microcentrifuge tube and then washed twice with 1× PBS (Phosphate buffer solution, Thermo Fisher Scientific). RLN buffer (Sigma-Aldrich) was used to disrupt the yolk sac releasing the cells into the buffer. This was then incubated on ice for 5 min and centrifuged. The resulting supernatant was collected and labeled as the cytosolic fraction. The pellet was washed twice with 200 µL of RLN buffer and finally collected and labeled as the nuclear fraction. RNA was extracted from the nuclear and cytosolic fractions of the embryos using the RNeasy mini kit from QIAGEN and was DNase (Sigma-Aldrich) treated to remove traces of genomic DNA. The quality of the RNAs extracted was detected on the RNA TapeStation using Agilent High Sensitivity RNA Screen Tape assay. Sequencing libraries were prepared using the TruSeq Stranded Total RNA library Prep kit with the Illumina Ribo-Zero rRNA removal kit (Human-Mouse-Rat). The quality check, library preparation, and sequencing were carried out by University of Birmingham's Genomics facility using Illumina NextSeq 500.

The RNA-sequencing reads obtained were mapped to the Zv9 genome assembly of zebrafish using STAR v2.6.0a (Dobin and Gingeras 2015) as explained earlier. Aligned reads were assembled into transcripts using StringTie (Pertea et al. 2015). Further, the Ballgown program (Pertea et al. 2016) was used to produce differential expression values (in transcripts per million [TPM]) in different stages of development. These expression values were used for all our downstream analysis.

### CAGE sequencing and analysis

CAGE library preparation was carried out using a modified cap trapping protocol (Carninci et al. 1996) for low quantity samples or LQ-ssCAGE protocol (Takahashi et al. 2020). RNA was extracted using the RNeasy mini kit from QIAGEN with 1 µg RNA per sample as starting material. We divided each sample into four parts, 250 ng of RNA to be pooled later after cDNA synthesis. Raw tags from CAGE sequencing were mapped using STAR aligner, and the resulting BAM files were used in the bioconductor package CAGEr for downstream analysis, including quality filtering, normalization, removal of the 5′ end G nucleotide that was added during the CAGE protocol (Haberle et al. 2015). The visualization of strand-specific CAGE tracks was carried out using “genomecov” command in the BEDTools package (Quinlan and Hall 2010).

### Statistical analysis

Significance levels (*P*-values) and sample sizes are provided in the text, figure legends, or indicated on the figures. Statistical analysis was performed using GraphPad Prism or as a part of the computational tools used. For measuring statistical significance, unpaired *t*-tests or χ^2^ tests were used to calculate *P*-value. *P*-values of <0.05 were considered significant.

## Data access

The RNA-seq and CAGE-seq data generated in this study have been submitted in the NCBI Gene Expression Omnibus (GEO; https://www.ncbi.nlm.nih.gov/geo/) under accession numbers GSE143208 and GSE144040, respectively.

## Supplementary Material

Supplemental Material
