# Supplementary material for: Antisense RNAs during early vertebrate development are divided in groups with distinct features
Source: Genome Res. 2021 Jun;31(6):995–1010. doi: 10.1101/gr.262964.120 (PMC8168585; doi:10.1101/gr.262964.120)
Supplement: Supplemental Material [file supp_31_6_995__DC1.html]

Antisense RNAs during early vertebrate development are divided in groups with distinct features — Antisense RNAs during early vertebrate development are divided in groups with distinct features — Supplemental Material 

# Antisense RNAs during early vertebrate development are divided in groups with distinct features

## Supplemental Material

- Supplemental\_figures.pdf
- Supplemental\_Table\_S1.xlsx
- Supplemental\_Table\_S2.xlsx
- Supplemental\_Table\_S3.xlsx
