# Supplementary material for: Antisense RNAs during early vertebrate development are divided in groups with distinct features
Source: Genome Res. 2021 Jun;31(6):995–1010. doi: 10.1101/gr.262964.120 (PMC8168585; doi:10.1101/gr.262964.120)
Supplement: Supplemental Material [file supp_gr.262964.120_Supplemental_figures.pdf]

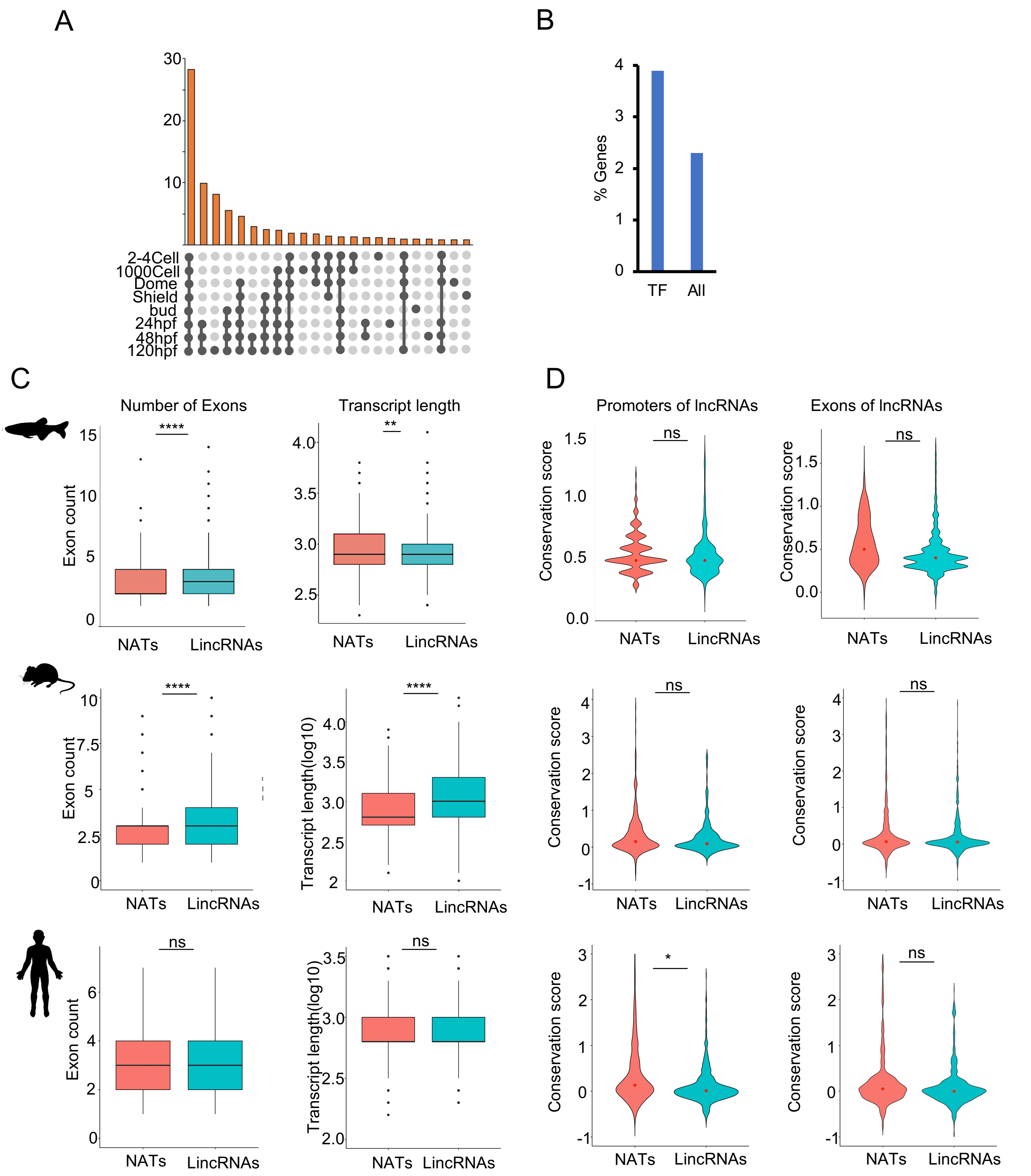

Supplemental Figure S1. (A) An UpSet plot showing showing the temporal stability of protein-coding genes. The percentage of mRNAs is shown on y-axis and the stages in which the mRNA is present is shown below x-axis. Filled circles represent the stages under consideration for the above bar. **(B)** A bar plot showing percentage of transcription factor (TF) genes and the percentage of all genes that overlap an annotated antisense RNA gene in zebrafish. **(C)** Boxplots showing differences in the number of exon and transcript length amongst zebrafish, mouse and humans. Each box shows median value (as horizontal lines) and extend from 25th to 75th percentile values for each group. The outliers are shown as dots. The significance values are as ns = non-significant, * P<0.05; **P<0.005; ***P<0.001; ****P<0.0001 (unpaired, two-tailed *t* test). **(D)** Violin plots displaying the conservation score at promoters and exons of NATs and lincRNAs in zebrafish, mouse and humans. Red dot indicates median value. The significance values are as ns = non-significant, * P<0.05; **P<0.005; ***P<0.001; ****P<0.0001 (unpaired, two-tailed *t* test).

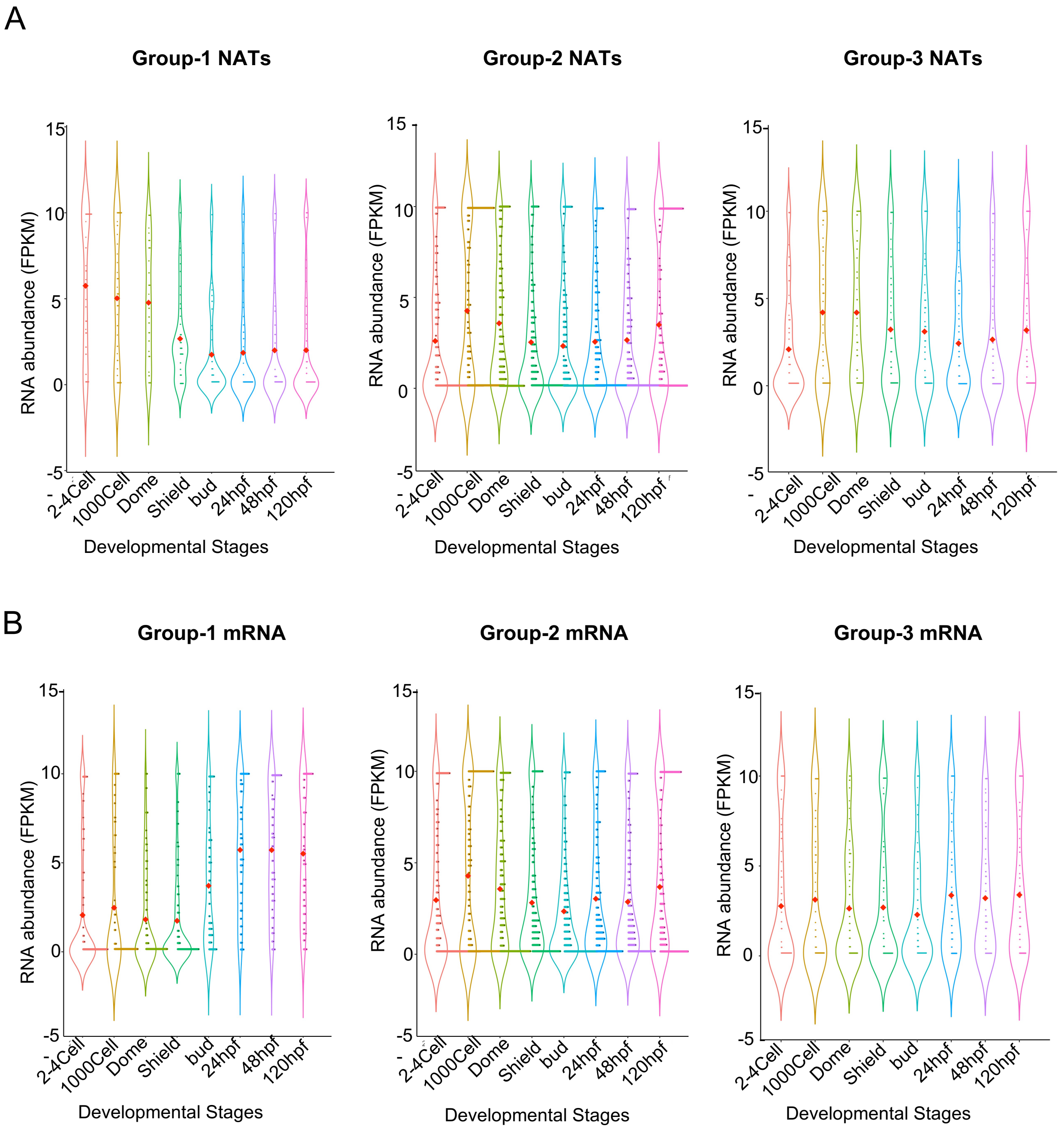

Supplemental Figure S2. (A) Violin plots showing the levels of NATs in group-1, group-2 and group-3 categories. **(B)** Violin plots showing the levels of overlapping mRNAs in group-1, group-2 and group-3 categories. The Y-axis represents the abundance of RNAs (FPKM) while the X-axis shows the stages of development. The red dot indicates the median value.

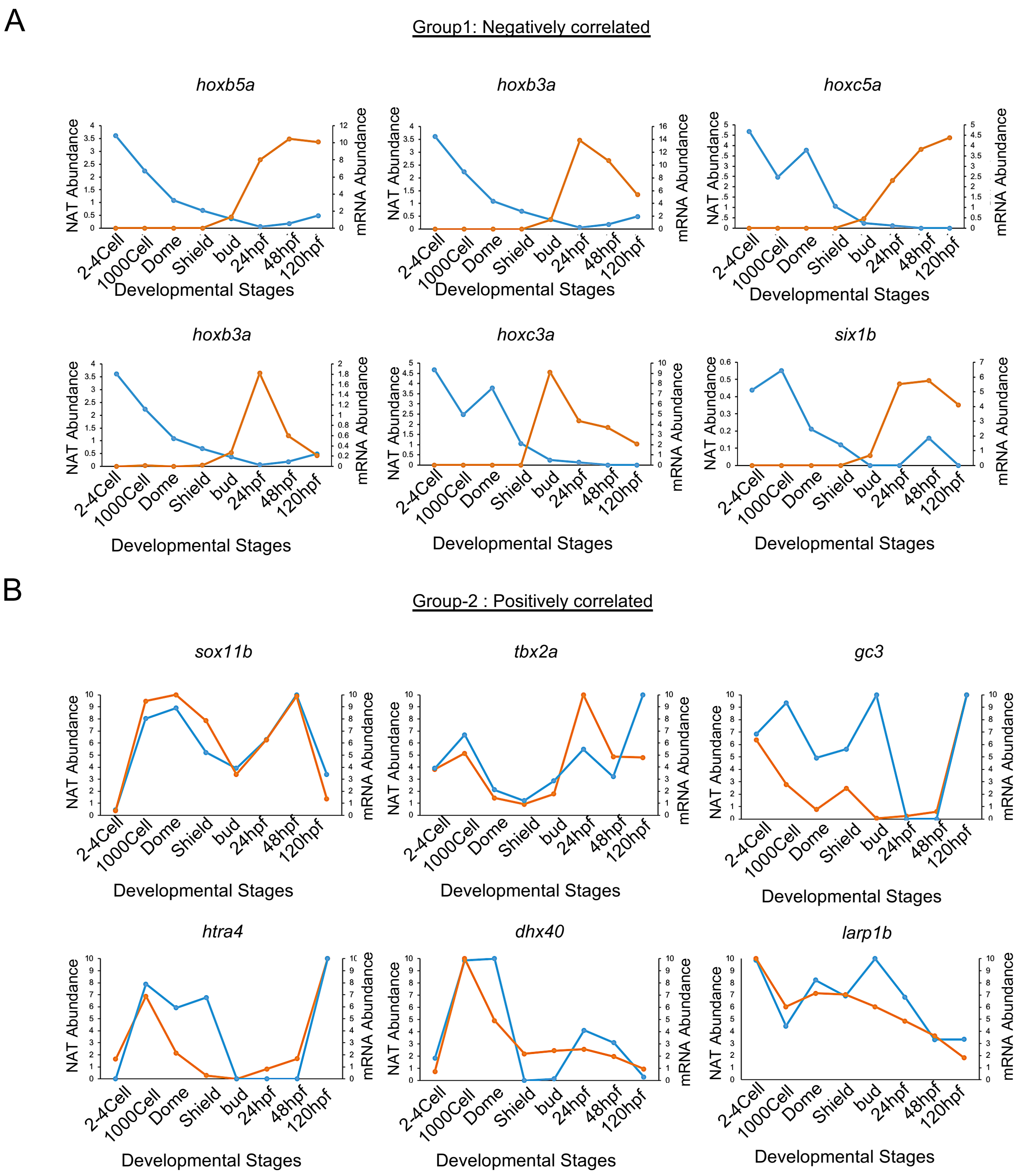

Supplemental Figure S3: (A) Individual examples of levels of NATs and overlapping mRNA in the group-1 category. (B) Individual examples showing RNA levels of NATs and mRNAs in group-2 category. The Y-axis represents the abundance of RNAs (FPKM) while the X-axis shows the stages of development.

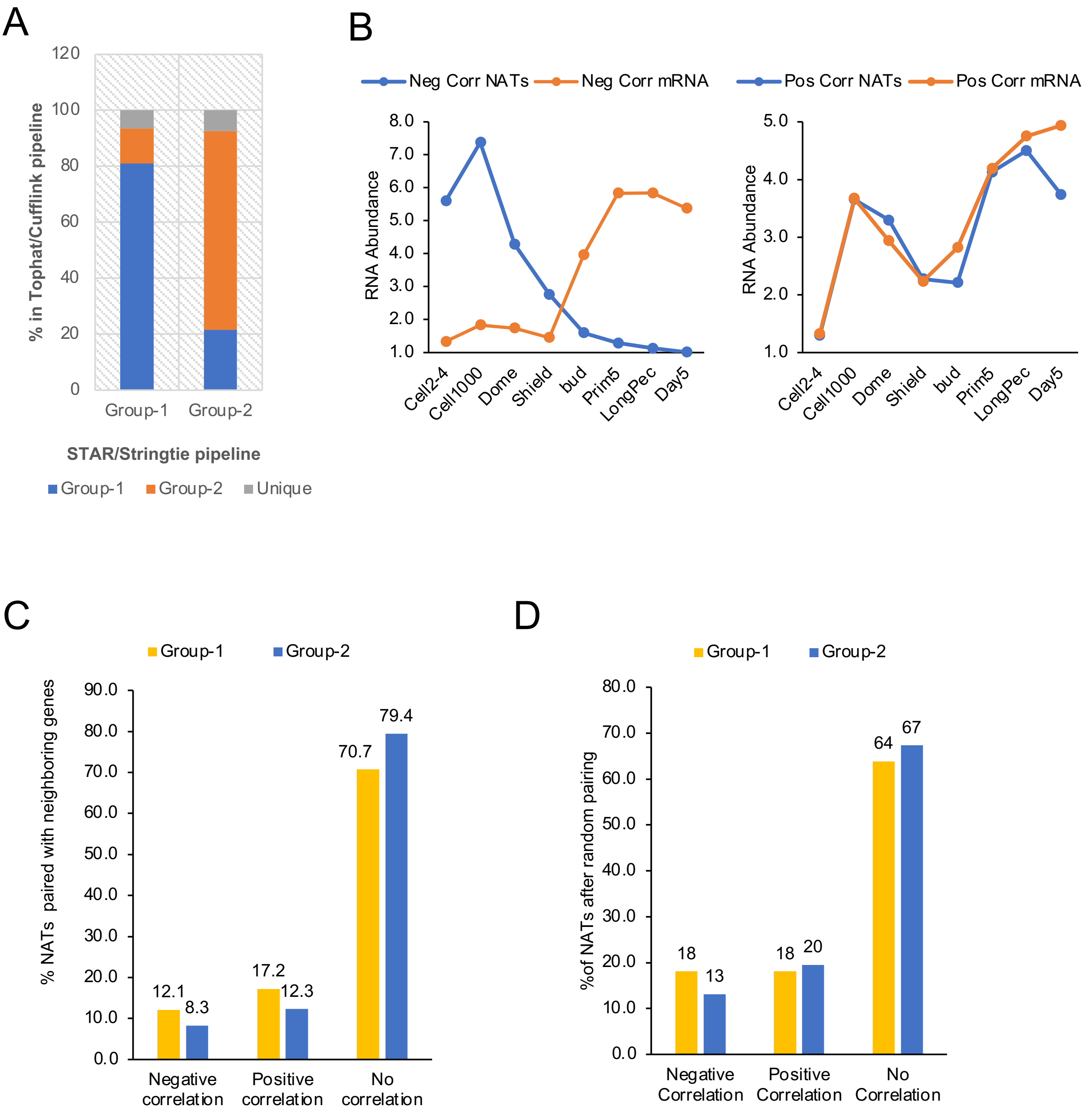

Supplemental Figure S4. (A) There are many RNA-seq alignment and analysis protocols available and each one of them can give slightly different estimates of RNA abundance. In order to confirm that the group-specific RNA abundance patterns that we observed (Figure 2B) were not a result of alignment protocol used, we additionally carried out the RNA-seq analysis using another frequently used alignment software STAR. A plot showing % overlap between STAR/Stringtie and Tophat/Cufflink RNA-seq analysis pipelines. The Y-axis represents the % of Group-1 (blue) and Group-2 (orange) NATs analysed by the Tophat/Cufflink protocol that were also categorized in the same group by STAR/Stringtie pipeline. **(B)** Line plots showing average abundance of NATs (blue) and overlapping mRNAs (orange), in the individual groups in the different stages of zebrafish development, calculated using STAR/Stringtie method. The abundance plots are comparable to the one in Figure 2B which are analyzed using TOPHAT/cufflink pipeline **(C)** Bar plot showing the % of group-1 (yellow) and group-2 NATs (blue) showing negative, positive and no correlations when paired with a neighboring protein-coding gene (not overlapping and +-4kb). We found that, when randomly paired, majority of group-1 (70.7%) and group-2 (79.4%) NATs show no correlation. **(D)** Bar plot showing the % of group-1 (yellow) and group-2 NATs (blue) showing negative, positive and no correlations when paired with randomly selected protein-coding gene from Zebrafish genome. Around 64-67% showed no correlation in the two groups of NATs upon random pairing.

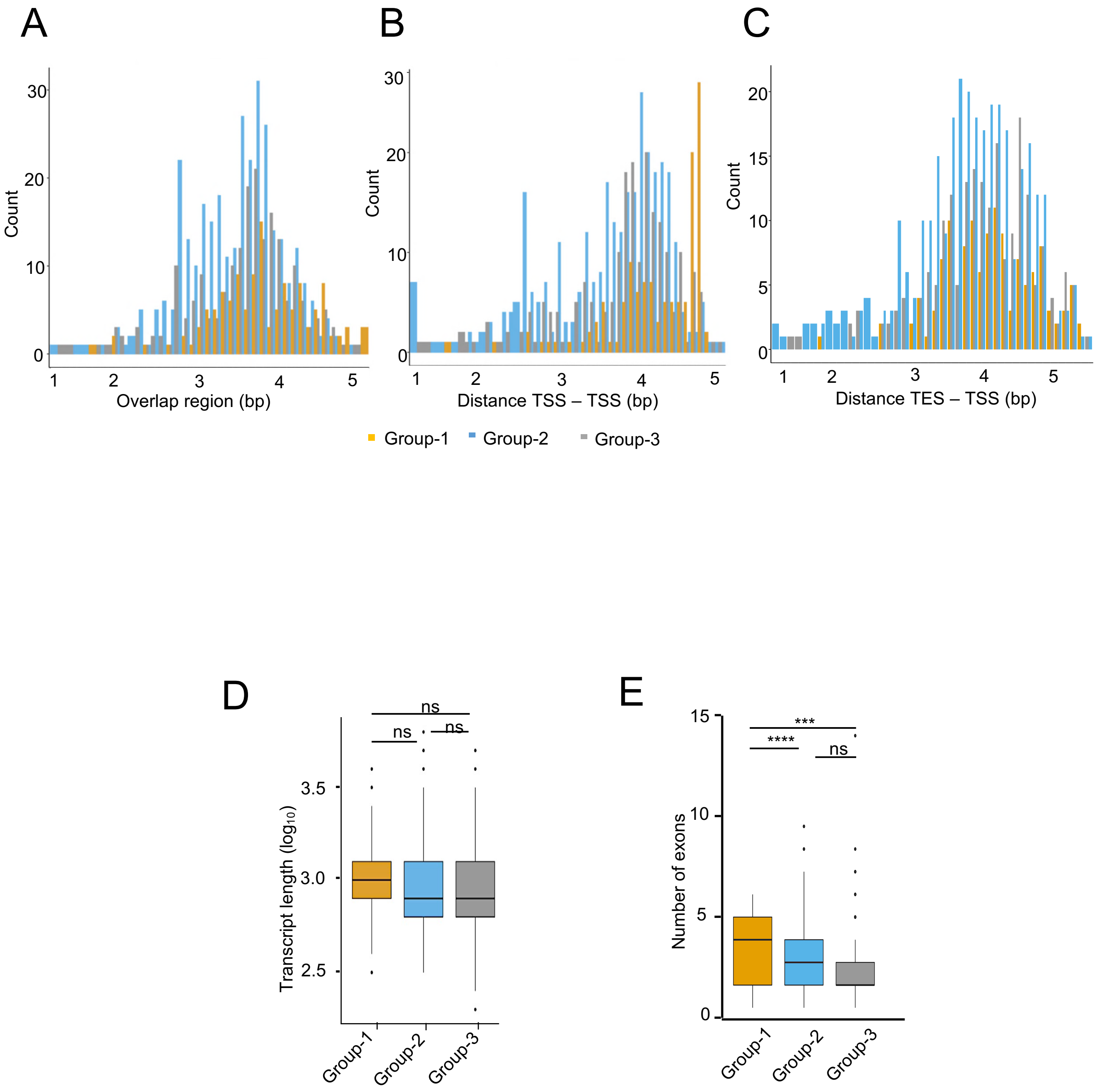

Supplemental Figure S5. **A)** A histogram showing the overlap region between the NATs and the overlapping mRNAs in the group-1 (yellow), group-2 (blue) and group-3 (grey). **(B)** A histogram showing the distance between the TSS of NATs and TSS of overlapping mRNA (log of distance in bp) in the three categories of genes as described in A. **(C)** A histogram showing the distance between the TES of AS and TSS of overlapping mRNA (log) in the three categories. The three histograms correspond to schematics and bar plots in main figure 3A, 3B and 3C. **(D) and (E)** Box plots showing differences in (D) transcript length and (E) the number of exons amongst the group-1, group-2 and group-3 NATs. Each box shows median value (as horizontal line) and extend from 25th to 75th percentile values for each group. The outliers are shown as dots. The significance values are as ns= non-significant, * P<0.05; **P<0.005; ***P<0.001; ****P<0.0001 (unpaired, two-tailed *t* test).

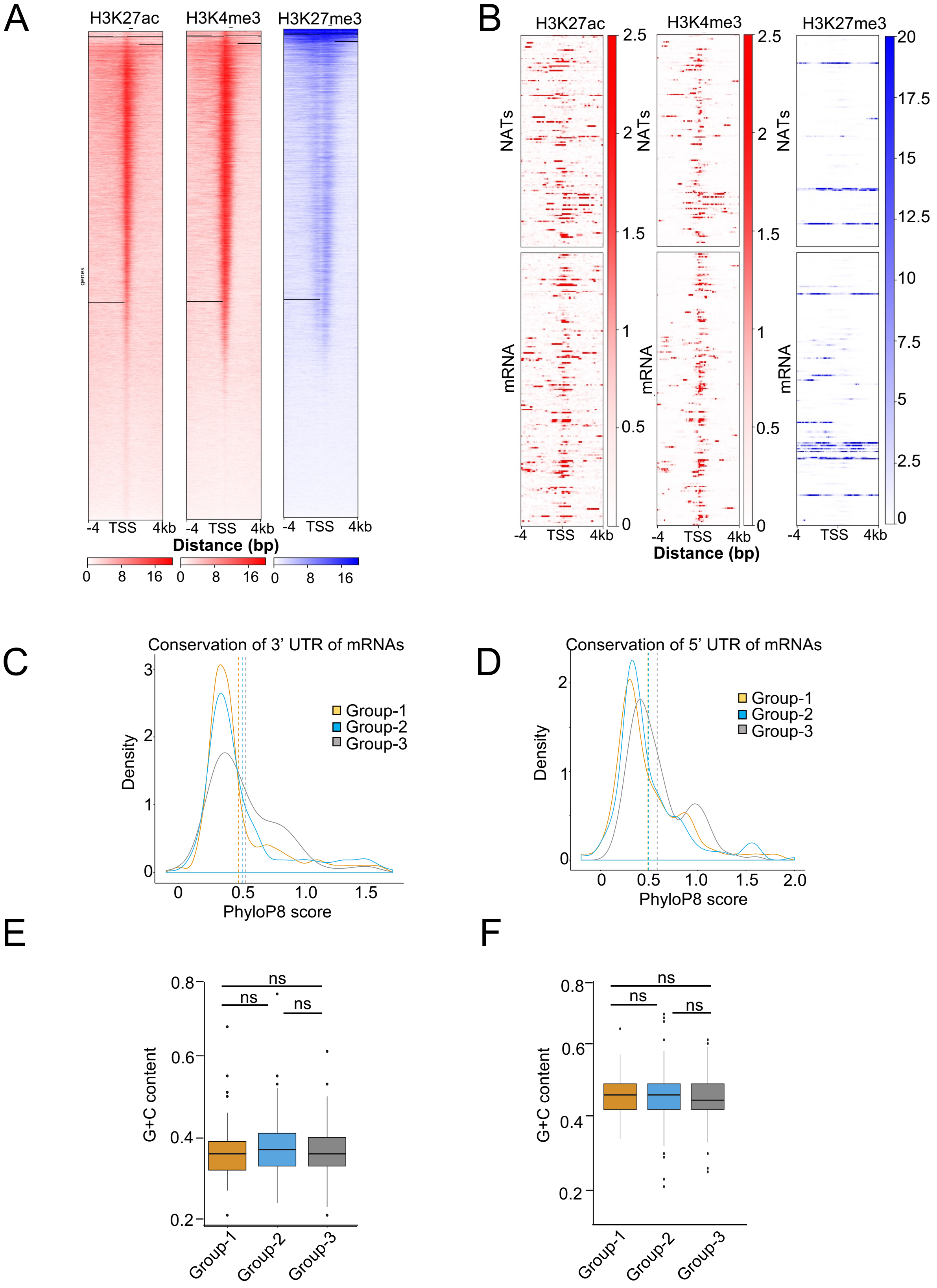

Supplemental Figure S6. (A) Heatmaps showing the enrichment of H3K27ac (red), H3K4me3 (red) and H3K27me3 (blue) ChIP-seq reads across the TSS of annotated mRNAs (±4kb). **(B)** Heatmaps showing the distribution of H3K2A27ac (red), H3K4me3 (red) and H3K27me3 (blue) ChIP-seq reads across the TSS of Group-3 NATs and mRNAs. **(C)** Density plots showing the conservation at 3' UTR and **(D)** 5' UTR (right) of protein-coding genes in the three groups. **(E)** Box plot highlighting G+C content at the 3' UTR and **(F)** 5' UTR of group-1, group-2 and group-3 mRNAs using EMBOSS-6.6.0. Each box shows median value (as horizontal lines) and extend from 25th to 75th percentile values for each group. The outliers are shown as dots. The significance values are as ns = non-significant, P > 0.05; * P<0.05; **P<0.005; ***P<0.001; ****P<0.0001 (unpaired, two-tailed *t* test).

A

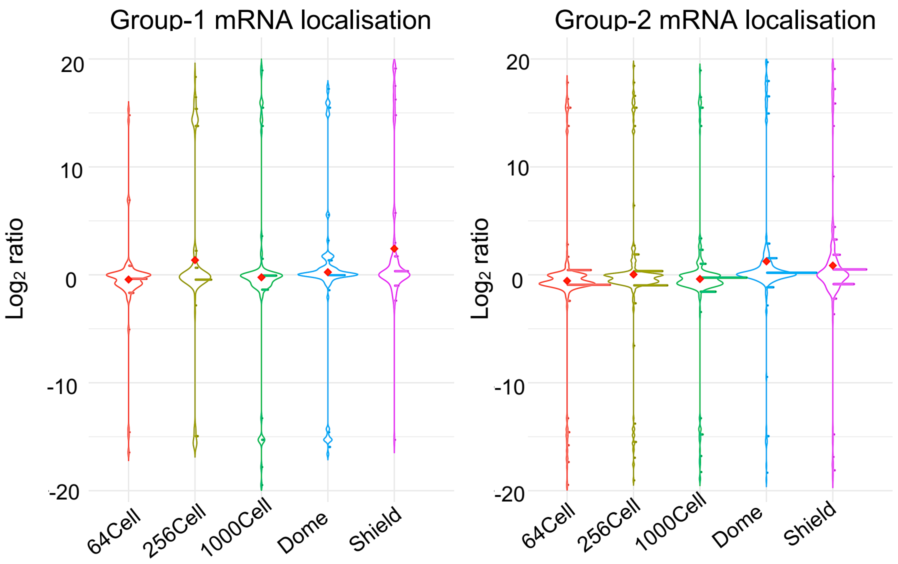

B

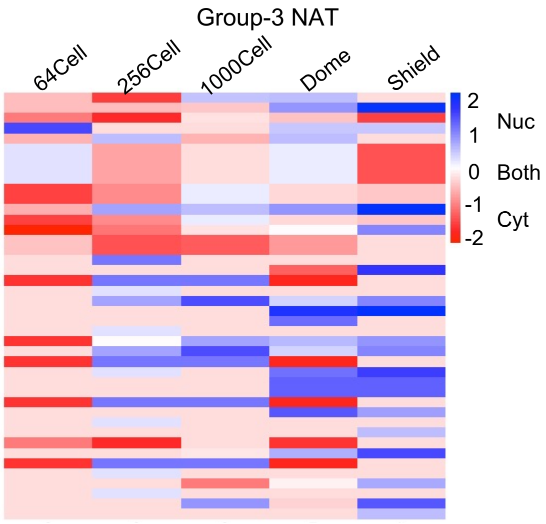

Supplemental Figure S7 (A) Violin plots showing the localization of group-1 and group-2 mRNAs during zebrafish development. The Y-axis displays the $\log_2$ ratio (Nuclear/Cytosolic) of RNA levels at different stages of development (x-axis) **(B)** A heatmap showing the localisation of group-3 NATs in the nuclear (blue) and cytosolic fraction (red) with zebrafish development. The group-3 NATs seem to be marginally (-0.5) more enriched in the cytosol compared to the nuclear fraction which is less compared to group-1 NATs (-1.0).
